# Cost Reduction of Inhaled Tobramycin by Use of Preservative-Free Intravenous Tobramycin Given via Inhalation

**DOI:** 10.3390/antibiotics5010002

**Published:** 2015-12-29

**Authors:** Timothy P. Gauthier, Justin Wasko, Nathan R. Unger, Lilian M. Abbo, Margaret Fernandez, Laura Aragon

**Affiliations:** 1 Department of Pharmacy, Miami Veterans Affairs Healthcare System, 1201 NW 16th Street, Miami, FL 33125, USA; 2 Department of Pharmacy, University of Minnesota Medical Center, 420 Delaware Street, Minneapolis, MN 55455, USA; Jwasko1@fairview.org; 3 College of Pharmacy, Nova Southeastern University, 11501 North Military Trail, Palm Beach Gardens, FL 33410, USA; NUnger@nova.edu; 4 Department of Medicine, Division of Infectious Diseases, University of Miami Miller School of Medicine, 2101 NW 117th Avenue, Miami, FL 33172, USA; labbo@med.miami.edu; 5 Department of Pharmacy, Jackson Memorial Hospital, 1611 NW 12th Avenue, Miami, FL 33125, USA; Mferandez7@jhsmiami.org (M.F.); lsmith5@jhsmiami.org (L.A.)

**Keywords:** inhaled antibiotics, pharmacy, pharmacotherapy

## Abstract

This study evaluates drug cost outcomes related to automatic therapeutic substitution of branded tobramycin solution for inhalation (TOBI^®^) with inhaled generic preservative-free intravenous tobramycin (PFIT). A retrospective single-center evaluation of inhaled tobramycin use from 2008 through 2012 was performed. Number of doses dispensed and acquisition costs were obtained. Hourly wage data was acquired, pharmacy production costs were estimated and total cost-savings calculated. Days of therapy (DOTs) were determined for each year. Quality assurance and safety data was collected. In 2008, TOBI^®^ drug costs and doses dispensed were $118,665 and 1769, respectively. Following implementation of the interchange in May 2009, TOBI^®^ utilization ceased. PFIT costs in 2010 through 2012 averaged $34,775 annually and TOBI^®^ cost-avoidance exceeded $94,000 annually when accounting for pharmacy production costs, which were determined to be at most $5.28 per dose. The maximum estimated pharmacy production cost ranged from $8812 to $11,299 annually. PFIT doses dispensed exceeded 1650 each year and annual DOTs ranged from 815 to 1069. The 40-month savings were calculated to be $374,706. Quality assurance and safety data identified one patient who refused PFIT due to odor complaints and one patient who was inappropriately administered a dose orally. Therapeutic substitution of TOBI^®^ with PFIT can produce immediate and sustained savings with an acceptable safety profile.

## 1. Introduction

Antimicrobial stewardship programs (ASPs) are interdisciplinary bodies that continuously strive to optimize patient care related to the use of antimicrobial agents [1]. In pursuing ASP development and sustainability, cost justification of allotted resources is essential. Automatic therapeutic substitution is one method utilized to reduce healthcare costs while sustaining patient quality of care. Through these policies, when a prescriber orders a high cost medication, an institutional protocol is in place directing the pharmacy to dispense a less expensive therapeutic equivalent. Such interventions can require minimal resources, produce significant cost-reductions and are typically approved by hospital policy makers (e.g., an inter-disciplinary Pharmacy and Therapeutics Committee). The literature infrequently describes outcomes related to automatic therapeutic substitution as an ASP strategy, yet these interventions may offer obtainable targets and require minimal resource investment. For example, replacing oral Vancocin^®^ with generic intravenous (IV) vancomycin prepared for oral administration has been previously reported to produce over $218,000 in annual cost-avoidance at a large medical center [2].

The national prevalence of inhaled tobramycin therapeutic interchange protocols is unknown; however, we are aware of several institutions that employ this strategy. In this substitution, the branded inhaled tobramycin product (TOBI^®^, Novartis Pharmaceuticals Corporation, East Hanover, NJ, USA) is interchanged with inhaled generic preservative-free IV tobramycin (X-Gen Pharmaceuticals Inc., Northport, NY, USA; PFIT) [3,4]. PFIT is made-to-order by hospital pharmacy sterile products personnel in accordance with standards issued by the United States Pharmacopeia chapter 797 [5]. The product is placed into a non-luer lock oral syringe and labeled “for inhaled use only”. Each dose is administered via nebulization by a respiratory therapist and the lack of a luer-lock prevents the solution from being inadvertently given via injection. A comparison of TOBI^®^ and PFIT is provided in Table 1.

**Table 1:** Comparison of TOBI^®^ and PFIT, inhaled tobramycin products [3,4].

Preservatives	TOBI^®^	PFIT
	No	No
Packaging	300 mg tobramycin plus 11.25 mg sodium chloride in 5 mL sterile water per ampoule	1.2 g power vial
Reconstitution	None Required	Under sterile conditions, pharmacy-reconstituted with 30 mL of 0.9% sodium chloride to a concentration of 40 mg tobramycin per mL
Dosing	300 mg (5 mL) inhaled twice daily	300 mg (7.5 mL) inhaled twice daily, dispensed in an oral syringe with a “for inhalation” auxiliary label
Stability	Per labeled package or more than 28 days at room temperature (up to 25° Celsius)	Reconstituted vial has a 24 h stability at room temperature (up to 25° Celsius) and 96 h stability under refrigeration (2° to 8° Celsius). PFIT doses are labeled with a 24-h expiration ^a^
Storage	May be stored in ADC	Due to 24-h expiration, limited ADC storage potential

ADC, automated dispensing cabinet; TOBI^®^, branded inhaled tobramycin product; PFIT, inhaled generic preservative-free intravenous tobramycin. ^a^ A conservative expiration of 24-h or less is assigned to all inhaled antibiotic products per pharmacy policy to ensure product integrity. Note that safety concerns have been identified with storage of inhaled colistin exceeding 24 h, but this has not been the case with PFIT [6].

Inhaled administration of injectable drug formulations is common practice [7]. For example, all inhaled colistin is prepared from the IV colistimethate sodium product. In the case of tobramycin, an IV product was exclusively used for inhalation prior to the approval of TOBI^®^ in 1998 [8,9,10,11,12]. Despite the availability of TOBI^®^, various preparations of tobramycin for inhalation continue to be used and studied worldwide including those containing sodium metabisulfite, EDTA or phenol as well as an antioxidant/preservative free formulation [13,14,15,16,17,18,19,20,21]. The therapeutic substitution of TOBI^®^ with PFIT is perceived as equivalent in regards to efficacy and safety, although data regarding the use of either product is limited to professional experience, clinical consensus and trials involving relatively small patient populations, mostly with cystic fibrosis (CF) [19,20,21]. Recently several other inhaled tobramycin products have become available. From the drug cost perspective, substantial price differences exist. Table 2 shows the average wholesale price for each inhaled tobramycin product [22].

**Table 2:** Cost-comparison of inhaled tobramycin products [22].

Product Name	Manufacturer	AWP Per Dose	AWP Date ^a^
TOBI^®^	Novartis^®^	$157.24	April 2014
Generic inhaled tobramycin	TEVA^®^	$128.77	November 2013
BETHKIS^®^	Cornerstone Therapeutics^®^	$121.61	May 2014
PFIT	X-Gen Pharmaceuticals^®^	$52.50	June 2012

^a^ AWP data is the most current available as of 6th November 2014. AWP, Average Wholesale Price; PFIT, inhaled generic preservative-free intravenous tobramycin.

Our institution is a large, academic-affiliated tertiary care hospital in Miami, Florida, USA. In May 2009, with support of the Pharmacy and Therapeutics committee, the ASP of our hospital implemented an automatic therapeutic substitution of branded TOBI^®^ with PFIT as a cost-savings initiative. The purpose of this study was to report drug cost outcomes related to this intervention. Our secondary aim was to identify any safety concerns following implementation of PFIT as the preferred product.

## 2. Results

### 2.1. Drug Usage Assessment

**Figure 1:**
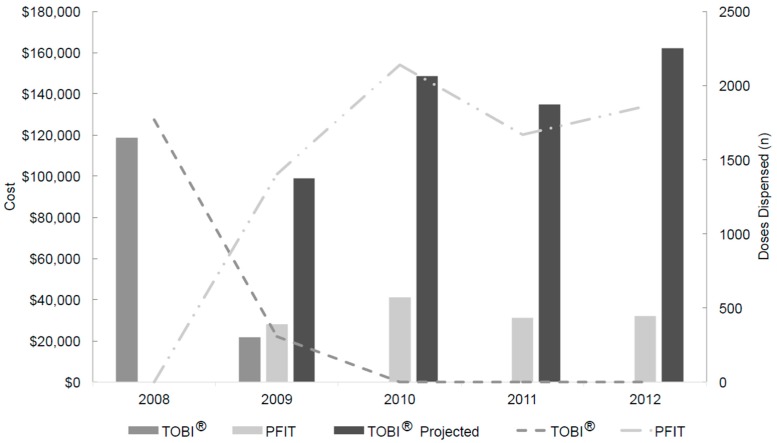
Inhaled tobramycin product utilization, actual costs and projected costs from 2008 through 2012, price-adjusted to 2013 US dollars ^a^. ^a^ Projected refers to the calculated cost of TOBI^®^ (based upon doses of PFIT dispensed and TOBI® contract pricing) post-intervention; TOBI^®^, branded inhaled tobramycin product; PFIT, inhaled preservative-free intravenous tobramycin.

From 1 January 2008 through 31 December 2012, 9146 doses of inhaled tobramycin (either product) were dispensed to a total of 486 patients and annual doses exceeded 1650 for all years (Figure 1). Following the intervention in May 2009, no further TOBI^®^ doses were dispensed or requested, while 7069 doses of inhaled PFIT were dispensed to a total of 348 patients. Inhaled tobramycin DOTs in 2008 through 2012 were 878, 843, 1069, 815 and 925, respectively.

### 2.2. Workload Evaluation

To prepare one dose of PFIT, under sterile conditions a pharmacy technician mixes the commercial 1.2 g preservative free preparation of tobramycin for IV use with 30 mL of normal saline for a final concentration of 40 mg/mL [4]. Next, 300 mg (7.5 mL) doses are drawn into single use 10 mL non-luer lock oral syringes and auxiliary labels “for inhalation only” and “refrigerate” are applied along with the product label. This process takes up to 5 min. After verification of the product by a pharmacist for accuracy (up to 30 s), it is stored under refrigeration with a labeled expiration of 24 h [4]. Lastly, the product is delivered to the necessary patient care area (up to 5 min). All hospital PFIT production is typically completed two times per day and distribution is commonly completed during routine floor deliveries. According to the May 2013 US Bureau of Labor Statistics, the median hourly wage for pharmacy technicians and pharmacists employed in a general medical and surgical hospital are $16.93 and $55.49, respectively [23,24]. The cost of non-drug supplies used during preparation and dispensing was estimated to be $2.00 per dose which in combination with hourly wage data, results in a maximum total non-drug cost of $5.28 per PFIT dose dispensed. Combining this data with the number of doses dispensed each year, the maximum cost of pharmacy production ranged from $8,812 to $11,299 annually.

### 2.3. Acquisition Drug Costs and Savings Determination

Following the intervention in May 2009, no further TOBI^®^ was purchased. Actual and projected drug costs are presented in Figure 1, using institution-specific data. Hospital costs for both drugs changed throughout the study period due to contracting agreements and market fluctuation. Including pharmacy overhead cost estimates, the mean 2010–2012 annual cost-avoidance was $103,789 (range $94,892 to $120,327). The total 40-month intervention period savings was approximately $374,706. Table 3 provides a comparison of the pre- and post-intervention periods.

**Table 3:** Comparison of the inhaled tobramycin automatic therapeutic substitution intervention, price-adjusted to 2013 US dollars.

Product	Production Cost Per Dose ($) ^a^	Pre-Intervention Period (January 2008 to May 2009)		Post-Intervention Period (May 2009 to December 2012)		Production Cost ($) ^c^	Cost Savings ($)
		Doses Dispensed (n)	Drug Cost ($)	Doses Dispensed (n)	Drug Cost ($) ^b^		
**TOBI^®^**	0	2077	140,402 (actual)	0	544,497 (projected)	0	-
**PFIT**	5.28	0	0 (actual)	7069	132,467 (actual)	37,324	374,706

^a^ Calculated using supplies, pharmacy technician and pharmacist time involved in the product preparation, verification and delivery; ^b^ Projected TOBI^®^ costs were calculated by multiplying TOBI^®^ institutional contract pricing by the number of dispensed PFIT doses; ^c^ Production cost was calculated by multiplying production cost per dose by number of doses dispensed.

### 2.4. Safety Assessment

Provider interviews and review of historical medication safety report data were completed in January 2013, revealing no concerning adverse events. During in-person interviews, providers noted awareness of the interchange and no controversy related to the intervention was identified. The quality assurance audit completed in 2013 focused on electronic records for non-pregnant, non-incarcerated adult inpatients who were not in a persistent vegetative state, concurrently receiving neuromuscular blocking agents or on inhaled hyper-tonic saline. Thirty-two patients who received PFIT were included. One patient refused PFIT secondary to odor complaints and no other unexpected events were found. Cough during PFIT administration was common (20 of 32 patients, 63%). For these patients, cough due to other inhaled medications was also common (13 of 20 patients, 65%). After application of the Naranjo algorithm to the patients with cough, 2 had *possible* and 1 patient had *probable* PFIT-associated cough. Additionally, one patient was found to have been incorrectly administered the solution via oral route. This patient experienced diarrhea, but had a complicated clinical course due to baseline illness severity and comorbidities. No other adverse effects were identified.

## 3. Discussion

Substitution of TOBI^®^ with PFIT resulted in an immediate and prolonged financial impact of at least $94,000 annually with minimal resource requirements and without identification of safety concerns. To our knowledge, this is the first report of ASP-generated cost savings resulting from the automatic therapeutic interchange of an inhaled antibiotic.

Antimicrobials may compose upwards of 30% of a hospital pharmacy budget and are historically one of the most costly therapeutic classes of drugs [25,26]. For an institution such as ours that spent over $4.6 million on antimicrobial agents in fiscal year 2012, these types of interventions are paramount as we seek to be financially responsible. Unfortunately, literature regarding the impact of ASP interventions on costs is limited, as demonstrated by data provided by the Centers for Disease Control and Prevention [27]. Our ASP continuously develops initiatives geared towards optimization of patient care and cost savings, with a particular interest for identifying interventions that produce results, but require limited resources. The term “low-hanging fruit” has been used to describe such interventions and the aforementioned use of IV vancomycin for oral administration is an example of this [2]. When assessing our intervention from the perspective that in 2012 we achieved savings of less than 3% of our 2012 fiscal year antimicrobial expenditures, one may conclude it to be a minor success. However, due to the absolute and continued financial impact, we consider this intervention to be a substantial success and suggest other ASPs consider this as a low-hanging fruit opportunity.

Examining potential pitfalls of an intervention is important and in this instance a further understanding of why the available products are considered therapeutically equivalent may be of value. For studies of all inhaled antimicrobials, those seeking to prove clinical outcome differences are many times confounded by a lack of consistency between drug delivery systems, the progressive nature of most pulmonary diseases and flawed endpoints [17,28]. Inherent heterogeneity introduced by a range of indications for therapy, concurrent inhaled medications, physiologic status, ventilator status, particle size, particle viscosity, and particle surface tension further impact these evaluations. In addition, physical properties such as osmolality, sodium content, pH, and the presence of preservatives may play a role. Factors such as these effect pulmonary penetration and as a result impact antimicrobial killing as well as the potential for adverse drug reactions. Thus, studies on this topic are complicated and the external validity of current publications is limited. In effect, quality assurance assessments and continued monitoring of the literature remains important.

Literature investigating a variety of inhaled tobramycin formulations have not found a difference in adverse drug reactions among any studied inhaled products, with cough and bronchospasm remaining the most common occurrences [14,15]. Thus, our finding of cough as a common occurrence was expected. Nikolaizik *et al.* compared tobramycin for inhalation with and without preservatives/antioxidants *vs* placebo and found no change in forced expiratory volume in one second (FEV1) or forced vital capacity (FVC) [18]. In a separate study, Nikolaizik *et al.* evaluated an IV preparation containing preservatives and antioxidants *vs* TOBI^®^ and also did not find differences in FEV1 and FVC [13]. In a study by Alothman *et al.*, patients identified as high risk (those with a positive response to bronchodilators and a history suggestive of asthma) were more likely to have bronchospasm irrespective of the tobramycin formulation, while low risk patients had a higher prevalence of bronchospasm with the preservative containing preparation, suggesting certain patients may possess inherent airway hypersensitivity [19]. After considering the available literature concomitantly with our study results, we believe the preservative-free formulation should continue to be utilized for this purpose.

There are limitations to our study. First, external validity may be influenced by institution-specific costs and product availability. Since intervention implementation, additional TOBI^®^ alternatives have become available. As a result, in mid-2014 we re-evaluated our inhaled tobramycin interchange, finding that a change in practice would not be financially advantageous and use of PFIT continues to produce substantial savings. To estimate potential institution-specific cost savings, communication with the pharmacy procurement coordinator is recommended. Second, we did not account for the additional respiratory therapist time the additional 2.5 mL of PFIT brings nor did we account for the cost of product refrigeration or drug wastage. Following a review of practices and resources at our institution, evidence suggesting these factors had an impact on our results was not found, but could not be substantiated by data and is an important limitation of our analysis. In regard to wastage, at an institution where the frequency of inhaled tobramycin use is low, in that the 1.2 g vial divided into four doses with a 24-h expiration would result in wastage, the option exists to produce one dose labeled with a 24 h expiration and then store the remainder of the multi-dose vial under refrigeration with a 96 h expiration for later use. Third, safety data acquired from the retrospective quality assurance audit was for a limited number of patients. A large prospective study of matched cohorts receiving PFIT and TOBI^®^ would be necessary to make a product comparison. Notably, the number of patients included in the analysis is comparable to many previous related publications on this topic [13,15,16,18,19,20,21]; Fourth, the concentration of PFIT differed from that of TOBI. While the total dose remained the same, the impact this different concentration could have had on efficacy or toxicity was not assessed. Fifth, interview data may be impacted by recall bias, but given the number of interviewees and continual relationship between ASP personnel and interviewees, we believe this to be of minimal consequence. Finally, it should be noted that the capacity to identify occurrences retrospectively is dependent upon available documentation. A prospective design for the toxicity evaluation would have produced results without this important limitation.

## 4. Methods

Pharmacy purchasing records and electronic medical records were accessed to acquire historical institutional drug costs and the number of inhaled tobramycin doses dispensed to hospitalized patients from 2008 through 2012. Days of therapy (DOTs) were calculated for each year to characterize annual drug consumption. To determine the pharmacy workload and production cost, pharmacy technicians and pharmacists were interviewed, supply costs were calculated and salary data was acquired from the United States (US) Bureau of Labor Statistics. The maximum potential dollar amount was selected for each step in the production process to ensure underestimation of production costs would not confound results. Quality control, staff training and wastage were considered for inclusion within the cost analysis, but based upon pharmacy personnel interviews were determined to be minimal and excluded. All reported costs were inflated to 2013 US dollars using the medical component of the Consumer Price Index [29]. Findings were combined to establish total cost savings and are presented using descriptive statistics. Note, institution-specific drug costs rather than average wholesale prices (noted in Table 2) were utilized for determining actual and projected financial results. Additionally, it should be noted that this was not a pharmacoeconomic analysis.

For the safety analysis, we evaluated the results of a quality assurance audit and applied the Naranjo algorithm to identify whether an adverse reaction was associated with PFIT [30]. We additionally interviewed numerous providers (e.g., respiratory therapists, clinical pharmacists, medication safety personnel) and reviewed historical medication safety report data. Questions posed to the interviewees investigated whether they were aware of the interchange, whether they were aware of any controversy regarding the interchange, and what their personal experiences were when working with inhaled tobramycin at our institution. This study was approved by the appropriate Institutional Review Boards.

## 5. Conclusions

Automatic therapeutic substitution of branded TOBI^®^ with PFIT produced an immediate and sustained financial impact totaling just under $375,000 over 40 months at our institution without identification of safety concerns.
